# Deep learning-based cell segmentation for rapid optical cytopathology of thyroid cancer

**DOI:** 10.1038/s41598-024-64855-2

**Published:** 2024-07-16

**Authors:** Peter R. Jermain, Martin Oswald, Tenzin Langdun, Santana Wright, Ashraf Khan, Thilo Stadelmann, Ahmed Abdulkadir, Anna N. Yaroslavsky

**Affiliations:** 1 Advanced Biophotonics Laboratory, University of Massachusetts Lowell, Lowell, MA USA; 2 Department of Radiation Medicine, MedStar Georgetown University Hospital, Washington, DC USA; 3 Centre for Artificial Intelligence, Zurich University of Applied Sciences, Winterthur, Switzerland; 4 Department of Pathology, UMASS Chan Medical School-Baystate, Springfield, MA USA; 5 ECLT European Centre for Living Technology, Venice, Italy; 6 University Hospital of Old Age Psychiatry and Psychotherapy, University of Bern, Bern, Switzerland; 7 Department of Dermatology, Massachusetts General Hospital, Boston, MA USA

**Keywords:** Thyroid cancer, Cytopathology, Fluorescence polarization, Methylene blue, Automated cell segmentation, Semantic segmentation, Optical imaging, Applied optics

## Abstract

Fluorescence polarization (Fpol) imaging of methylene blue (MB) is a promising quantitative approach to thyroid cancer detection. Clinical translation of MB Fpol technology requires reduction of the data analysis time that can be achieved via deep learning-based automated cell segmentation with a 2D U-Net convolutional neural network. The model was trained and tested using images of pathologically diverse human thyroid cells and evaluated by comparing the number of cells selected, segmented areas, and Fpol values obtained using automated (AU) and manual (MA) data processing methods. Overall, the model segmented 15.8% more cells than the human operator. Differences in AU and MA segmented cell areas varied between − 55.2 and + 31.0%, whereas differences in Fpol values varied from − 20.7 and + 10.7%. No statistically significant differences between AU and MA derived Fpol data were observed. The largest differences in Fpol values correlated with greatest discrepancies in AU versus MA segmented cell areas. Time required for auto-processing was reduced to 10 s versus one hour required for MA data processing. Implementation of the automated cell analysis makes quantitative fluorescence polarization-based diagnosis clinically feasible.

## Introduction

Thyroid cancer is the 9th most common malignancy worldwide^1^. Approximately 44,000 new cases are expected to be diagnosed this year in the United States^1,2^. Besides, studies show that over 60% of adults will develop one or more benign thyroid nodules during their lifetime^3^. Benign nodule growth can be influenced by various health factors (e.g., age, genetics, hormonal changes, etc.) and only about 1% will result in thyroid disease^4^. Prognosis for malignant thyroid nodules largely depends on the type of cancer, tumor size and stage at diagnosis, as well as subsequent medical intervention^5,6^. Therefore, early and accurate differentiation of cancerous versus non-cancerous neoplasms is imperative for guiding clinical management of thyroid tumors^7,8^.

Fine-needle aspiration (FNA) cytopathology is an established clinical method for detecting thyroid cancer^9–14^. According to the Bethesda System for Reporting Thyroid Cytopathology (TBSRTC), thyroid FNAs are divided into six categories: (I) non-diagnostic, (II) benign, (III)–(V) indeterminate, which include atypical, undetermined, and suspicious for malignancy specimens, and (VI) malignant^15–19^. Definitive assessment of cytologically indeterminate samples (TBSRTC III–V), which represent 40% of thyroid FNA cases, requires histological evaluation of surgical specimens following lobectomy or complete thyroidectomy^20,21^. Surgical complications such as laryngeal nerve paralysis (5–11% of patients) or hypocalcemia frequently occur (20–30%)^21–23^. In all cases where surgical resection is completed, patients must be subjected to hormone replacement therapy for the remainder of their lifetime^23,24^.

Recently, molecular genetic testing has been implemented for the cytologically indeterminate thyroid nodules^25,26^. These assays may be able to detect malignant thyroid cells, determine the subtype of thyroid cancer, and/or provide information concerning the disease prognosis by identifying specific chromosomal abnormalities or gene mutations^27,28^. While some reports have indicated that genomic profiling can boost diagnostic accuracy of thyroid FNA^28^, others demonstrated low specificity (25%)^28,29^ and positive predictive value (35%)^29,30^. Moreover, molecular testing requires a dedicated FNA specimen that can be interpreted only in the context of a patient’s complete clinical workup. Widespread use of molecular testing has been hampered by substantial costs, protracted turnaround times, and requirements for complex laboratory facilities and equipment^31^. Thus, development and clinical implementation of accurate, quantitative, and economical methods for diagnosing thyroid neoplasms are imperative for addressing cytologically indeterminate thyroid nodules.

Methylthioninium chloride, commonly referred to as methylene blue (MB), is a positively charged, cytological stain approved for human use by the Food and Drug Administration (FDA) of the United States. Studies examining the use of MB as an exogenous contrast agent have demonstrated its affinity to malignant cells and tissues^32–37^. More recently, MB fluorescence polarization (Fpol) was proposed as a promising tool for cancer detection^38–41^. Fluorescence polarization is an optical technique that quantifies the degree of polarization of fluorescence emission relative to the polarization of incident light^42^. Whereas Fpol values are sensitive to factors that limit the rotational motion of the fluorophore during the lifetime of the excited state (e.g., increased local viscosity or binding), they are much less influenced by the underlying optical properties of cells and tissues as compared to fluorescence emission.

Previously, we assessed MB Fpol of thyroid FNA specimens in a double-blind, multi-institutional clinical study^43^. We demonstrated that MB Fpol is significantly higher in cancerous versus non-cancerous cells, including specimens from the cytologically indeterminate categories. However, clinical translation of the technology is hindered by manual data processing, which makes it expensive, labor intensive and time consuming. Employing automated cell segmentation and image processing holds the potential to make Fpol cytology faster and would allow its deployment in settings where no specifically trained personnel are available. In supervised training, an artificial intelligence (AI) system can learn from examples and train an automated algorithm to process new images comparable to how a human expert would do it^44–46^.

In this study we developed and evaluated a verifiable and trustworthy automated image analysis pipeline for segmenting clinically relevant cell areas and facilitating the Fpol data analysis. The goal of the project was to demonstrate feasibility of accelerating Fpol data processing, confirm diagnostic robustness of the Fpol method, and facilitate clinical translation of this promising new tool for quantitative cancer diagnosis.

## Results

### Performance of the U-Net model

**Figure 1:**
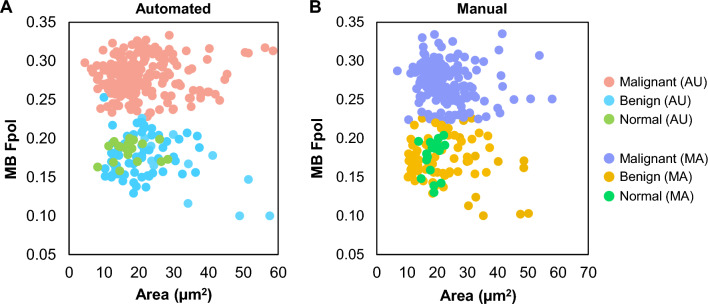
Scatter plot of MB Fpol value versus cell area (µm^2^). Circles represent cells in the test samples processed using (**A**) automated and (**B**) manual methods.

Cellular level MB Fpol values obtained by the U-Net-based automated (AU) cell segmentation are compared to manual (MA) data processing in Fig. 1. The scatter plots display all cells in the test set processed with AU (Fig. 1A) and MA (Fig. 1B) methods. The cells are grouped into three diagnostic categories: (i) malignant (papillary thyroid carcinoma, PTC; follicular thyroid carcinoma, FTC; medullary thyroid carcinoma, MTC), (ii) benign (follicular thyroid adenoma, FTA; multinodular goiter, MNG), and (iii) normal. Each cell is characterized by its size, calculated as region area, and the corresponding MB Fpol value. In total, 601 cells were analyzed from the test set, including 426 matched cells and 175 unmatched cells. Unmatched cells included 125 cells selected only by the model, and 50 cells chosen solely by the expert. The model selected 15.8% more cells than the human operator. Segmented areas were generally smaller with the AU method (e.g., AU area was smaller in 75% of matched cells); however, the range of Fpol values for matched and unmatched AU cells were very similar to that of the MA data. In Fig. 1A (data processed by the U-Net), the range of Fpol values were 0.228–0.333 (malignant cells), 0.100–0.253 (benign cells), and 0.158–0.200 (normal cells). In Fig. 1B (data processed manually), the ground truth cellular Fpol values ranged between 0.224–0.335, 0.100–0.226, and 0.130–0.204 for malignant, benign, and normal groups, respectively. In both methods cancerous cells are clustered at higher Fpol values, while non-cancerous cells (benign and normal) are distributed across lower Fpol values.

Applying the trained U-Net model to the test set and running the morphological post-processing operations and computation of Fpol values required less than 10 s per sample on a laptop equipped with an Intel i9-12900H (Intel Corporation, Santa Clara, CA) without any need for specialized hardware such as a high-performance graphics processor unit (GPU) or AI accelerator. In contrast, manual data processing required at least one hour to draw all the segmentation masks and perform Fpol calculations for each sample.

### Representative cases

The MB Fpol technology with automated image processing may provide an accurate, clinically viable tool for differentiating cytologically indeterminate thyroid nodules as cancerous or non-cancerous, as highlighted by the following cases selected from the study test set.

#### Malignant specimen (TBSRTC III)

**Figure 2:**
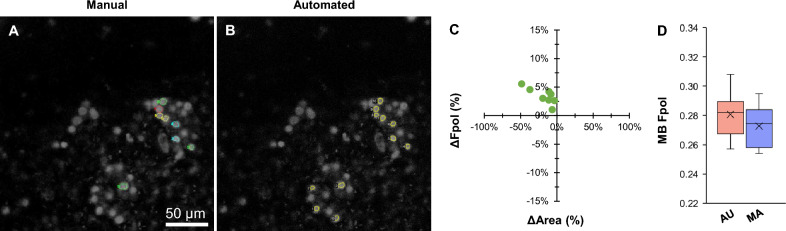
Representative data for malignant PTC cells (sample #6). MB fluorescence emission image with cells (**A**) traced by the human expert and (**B**) segmented by the model. (**C**) Scatter plot of percent difference in MB Fpol value versus cell area (µm^2^) for matched cells selected in both manual and automated methods. (**D**) Box plot of MB Fpol values of all cells from each method. Boxes extend from the first to the third quartiles. Black cross marks and horizontal lines represent the mean and median values, respectively. Vertical lines indicate 1.5 times the interquartile range from each box end.

Figure 2 displays a cancerous aspirate (sample #6) that yielded indeterminate results using conventional cytopathological evaluation. The nodule was categorized as TBSRTC III, and histopathology determined the definitive diagnosis as follicular variant of PTC. The MB fluorescence emission images in Fig. 2A,B display cytomorphology, including sheets of clustered PTC cells with nuclear crowding and enlargement. Figure 2A presents cells annotated by manual segmentation (multi-color regions), while in Fig. 2B the cells were segmented by the U-Net (yellow regions). Manual selection yielded 8 cells. The model segmented 11 cells, including all those selected manually, plus 3 additional unmatched cells (AU #0, #1, and #2). Detailed data for all the cells, including MB Fpol values and region areas, demonstrate good agreement between the AU and MA methods (Supplementary Table S1). Figure 2C displays a scatter plot of percent difference in Fpol versus cell area, for the matched cells selected automatically and manually. Overall, the Fpol values differed by 1.1–5.6%, whereas segmented areas varied between − 48.6 to − 3.2%. Larger differences in Fpol correlated with greater discrepancies in cell area (e.g., in MA #6, which corresponds to AU #8, there were variations of 5.6% and − 48.6% in Fpol and area, respectively). In Fig. 2D, the box plot shows the distribution of Fpol values. The range of Fpol values obtained via auto-segmentation (0.257–0.308) was comparable to manual tracing (0.254–0.295). Average MB Fpol measured automatically was 0.281 ± 0.015, whereas manually it was 0.273 ± 0.015 (difference of 2.8%).

#### Benign specimen (TBSRTC III)

**Figure 3:**
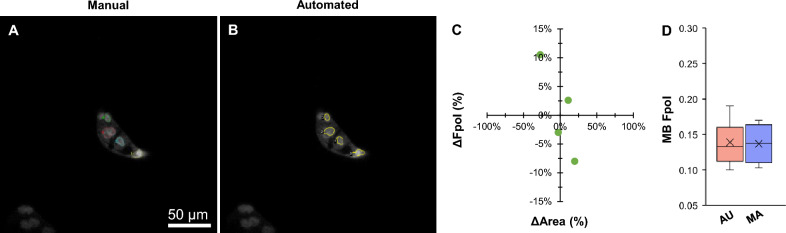
Representative data for benign FTA cells (sample #7). MB fluorescence emission image with cells annotated by (**A**) manual and (**B**) automated methods. (**C**) Percent difference in MB Fpol versus cell area (µm^2^) for matched cells. (**D**) Distribution of MB Fpol values.

Figure 3 presents an indeterminate TBSRTC III thyroid nodule (sample #7). The final diagnosis of benign FTA was determined using histopathology. In Fig. 3A,B, MB fluorescence emission images display clustered cells with small round nuclei and smooth nuclear margins. The same 4 cells were selected by AU and MA-based segmentation methods (Supplementary Table S2). In comparison, differences in Fpol values ranged between − 8 to 10.5%, and differences in cell areas differed by − 27.2 to 19.9% (Fig. 3C). Larger variations between Fpol values correlated with larger deviation in cell area. For example, MA #4 and corresponding AU #3 varied by 10.5% (Fpol) and − 27.2% (area). In Fig. 3D, average MB Fpol values were 0.139 ± 0.039 (range: 0.100–0.190) and 0.137 ± 0.034 (0.103–0.170) for AU and MA methods, respectively (difference of 1.4%).

#### Malignant specimen (TBSRTC IV)

**Figure 4:**
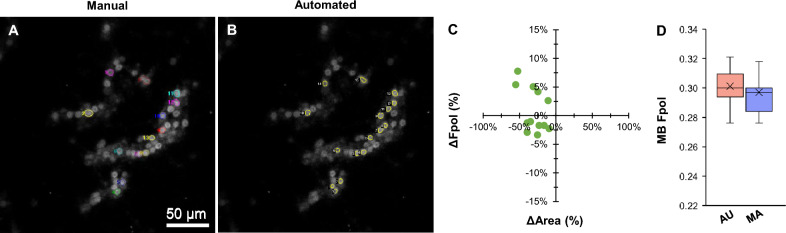
Representative data for malignant FTC cells (sample #5). MB fluorescence emission image with cells annotated by (**A**) manual and (**B**) automated methods. (**C**) Percent difference in MB Fpol versus cell area (µm^2^) for matched cells. (**D**) Distribution of MB Fpol values.

Figure 4 shows results for a cancerous FNA (sample #5) that was categorized as TBSRTC IV. Histopathology revealed the final diagnosis as poorly differentiated FTC. The MB fluorescence emission images in Fig. 4A,B show microfollicles with non-specific nuclear atypia, overlapping, and crowding. Manual segmentation yielded 13 cells for analysis, whereas the model segmented 16 cells, including 12 of those selected manually, plus 4 additional unmatched monomorphic cells with a round shape (AU #1, #8, #11, and #14). Detailed data for all cells are provided in Supplementary Table S3. In Fig. 4C, differences in Fpol values and segmented areas varied between − 3.3 to 7.8% and − 9.3 to − 55.2%, respectively. AU-derived Fpol values ranged from 0.276 to 0.321, while MA calculated Fpol values ranged from 0.276 to 0.325. Average MB Fpol of the samples obtained using both methods (AU: 0.301 ± 0.012; MA: 0.297 ± 0.014) agreed within 1.4% (Fig. 4D).

### Statistical assessment

**Figure 5:**
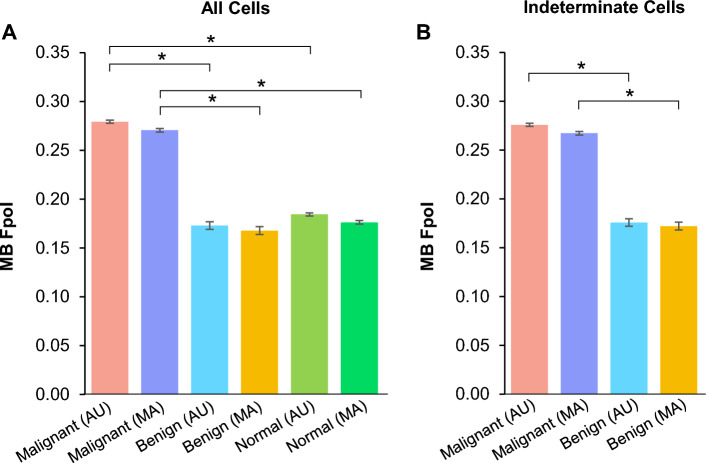
Average MB Fpol value of cells in the test samples processed using automated and manual methods for (**A**) all cells and (**B**) indeterminate cells. The cells were grouped by histological diagnosis: malignant (PTC, FTC, and MTC), benign (FTA and MNG), and normal. Error bars represent one standard deviation calculated over all cells in the respective category. *p < 0.0001.

Figure 5 shows average MB Fpol for the test samples where cells were grouped based on data processing technique (AU or MA), diagnostic classification (malignant, benign, or normal), and/or indeterminate cytology category. In Fig. 5A, the average MB Fpol value of all malignant cells (PTC, FTC, and MTC) determined by AU method was 0.279 ± 0.002, whereas with MA technique it was 0.270 ± 0.002 (difference of 3.2%). All benign cells (FTA and MNG) had an average Fpol value of 0.174 ± 0.004 and 0.168 ± 0.004 using AU and MA methods, respectively (difference of 3.4%). In all normal cells, the AU-derived average Fpol value was 0.184 ± 0.003, while that from the MA approach was 0.176 ± 0.005 (difference of 4.3%). Figure 5B shows average MB Fpol values for cells from the indeterminate cytology categories. In malignant cells, average Fpol values were 0.276 ± 0.002 (AU) and 0.267 ± 0.002 (MA) (difference of 3.3%), whereas for benign cells the average values were 0.176 ± 0.004 (AU) and 0.172 ± 0.003 (MA) (difference of 2.3%). Statistical analysis revealed that MB Fpol was significantly elevated in malignant versus benign (*p* < 0.0001) and malignant versus normal (*p* < 0.0001) cells in both AU and MA data sets. Importantly, there were no significant differences (*p* > 0.01) in MB Fpol values obtained via AU versus MA processing methods.

## Discussion

This is the first feasibility study that evaluated utility of automated segmentation for thyroid cancer detection at the cellular level using Fpol imaging. Our published work has demonstrated that MB Fpol provides a robust and reliable metric for quantitative differentiation of thyroid cancer in single cells^43^. However, the previously presented methodology required manual cell selection, segmentation, and Fpol calculation by expert microscopists and pathologists. In this contribution, we introduced and evaluated the U-Net-based model for rapid automated detection, segmentation, and Fpol evaluation of diagnostically relevant cells in thyroid fine-needle aspirates. The results (Fig. 1) demonstrate that automated patterns of cell selection and segmentation agreed well with expert hand tracing performed according to requirements and standards determined by pathologists. The developed model reduced time required for the analysis of one sample from 1 h to approximately 10 s and yielded Fpol values that are not statistically different (Fig. 5) from those obtained by manual assessment for all cell types and diagnoses investigated.

On average, cell areas segmented by the U-Net were 6% smaller as compared to the manually selected regions. There are several reasons for the model to underestimate the cell extent. First, the training set contoured by experts intentionally included cells with conservative boundary delineations. Due to the quantitative nature of Fpol image analysis, it is imperative to avoid inclusion of background pixels that could impact intensity measurements and lead to the wrong diagnostic classification. Second, as MB is a nuclear stain, cell nuclei are brighter in fluorescence emission images relative to the cytoplasm and other intracellular organelles. As a result, the model may preferentially select a smaller sub-region of some cells. In contrast, in certain cases the model grouped multiple cells into one large region, most notably when the image frame contained clustered or overlapping cells. Both these shortcomings can be addressed by optimizing sample preparation, staining, and image acquisition techniques, as well as utilizing computer vision methods for improving image quality, augmentation, and algorithmic modifications. The morphological operations removed spurious pixels that were segmented as cells but may have added some pixels to some cells. However, because the operations were closing in nature and the segmented areas were largely convex, the overall outlines of the cells did not change. Nevertheless, different parameters of the morphological operations may slightly impact the segmented areas. Importantly, despite these challenges, AU-derived Fpol values accurately distinguished malignant thyroid cells from benign or normal (Fig. 5). In addition, the model segmented 15.4% more cells than the human operator. All of these cells aligned with standard of care selection parameters and returned Fpol values consistent with the diagnosis of the respective FNA. Despite observed discrepancy in cell selection and systematic under-segmentation, the diagnostically relevant Fpol values were consistent with those from the manual segmentation. This furthermore underlines the robustness of the quantitative Fpol approach.

The intermediate result from the AI, which is the segmentation masks, depends on the architecture, the training procedure, the training data, and the post-processing such as the morphological operations. With changes to some of these aspects, the overlap accuracy may increase, but the consistency in Fpol value may not increase proportionally. Therefore, although there is room for improving the segmentation algorithm and possibly the manual annotation, we refrained from experimenting with additional class-preserving augmentations such as pixel-wise noise and elastic deformation to focus on the conceptual advancement of automating extraction of Fpol values.

In cytopathology, the Bethesda system yields high diagnostic sensitivity/specificity (> 90%) for distinctly benign (TBSRTC II) or malignant (TBSRTC VI) thyroid tumors. However, diagnosing nodules that fall within the indeterminate categories (TBSRTC III–V) remains a significant clinical challenge. Therefore, we specifically targeted these categories when selecting the test data set. Specifically, out of twelve indeterminate samples, eleven were included in the test set and one was used for training. The results summarized in Fig. 5B demonstrate statistically significant separation between Fpol values of malignant and benign indeterminate cells obtained utilizing AU segmentation, whereas the results obtained using AU and MA segmentation were not statistically significant. Quantitative results demonstrated that AU evaluations can rapidly and accurately differentiate cancerous versus non-cancerous indeterminate thyroid nodules (Figs. 2, 3, 4). Malignant PTC and FTC cells exhibited significantly higher (*p* < 0.0001) MB Fpol relative to benign FTA cells. These findings underscore specific applications where automated Fpol cytology could improve upon conventional FNA assessments. For example, in the evaluation of follicular thyroid lesions, FNA cannot currently distinguish FTA versus FTC due to morphological similarities between the cells. Interestingly, the results presented in Fig. 3 show that Fpol was capable of diagnosing FTA using the images of just four cells, as compared to at least sixty cells required for traditional cytological evaluation. Generally, these types of cases may be deemed suspicious for a follicular neoplasm and cannot be reliably determined as malignant or benign by FNA alone. Instead, histological review of surgical specimens would be needed to identify evidence of capsular invasion, vascular invasion, or invasion of the adjacent parenchyma. In contrast, AU-derived Fpol measurements could reduce uncertainty and deliver a prompt, accurate diagnosis directly following the FNA acquisition.

**Figure 6:**
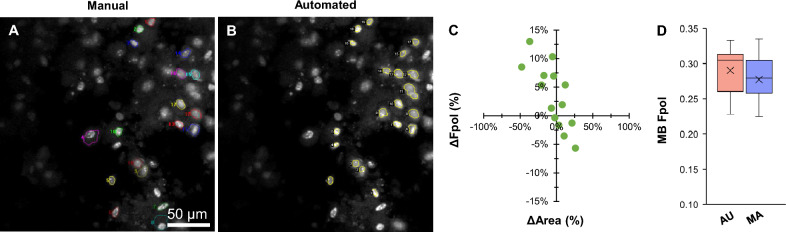
Representative data for malignant MTC cells (sample #10). MB fluorescence emission image with cells annotated by (**A**) manual and (**B**) automated methods. (**C**) Percent difference in MB Fpol versus cell area (µm^2^) for matched cells. (**D**) Distribution of MB Fpol values.

The AI model was trained using six samples, including three with cancerous PTC cells, which is the most common subtype of thyroid cancer (80–85% of cases). Other thyroid cancers, such as FTC (10–15% of cases) or MTC (4–10% of cases), were not included in the training set for this feasibility study. Nonetheless, the U-Net overcame limitations and accurately processed FTC and MTC cells based on morphological features learned from other training samples. While this kind of generalization is expected from deep learning models to some extent, more pronounced differences between samples observed during training and evaluation must be accounted for with specialized domain adaptation methods^47,48^. Automated results for FTC revealed highly elevated MB Fpol values in good agreement with the ground truth (Fig. 4). There was one MTC case in the test set. Results for this aggressive thyroid cancer are shown in Fig. 6. The ground truth had 18 cells (Fig. 6A) and the model segmented 20 cells (Fig. 6B) including 14 cells selected by the expert, plus 6 additional unmatched cells (Supplementary Table S4). The differences in Fpol values and cell areas varied from − 5.7 to 13.0% and − 47.8 to 26.1%, respectively (Fig. 6C). Despite lack of MTC cells in the training set, average MB Fpol values (AU: 0.288 ± 0.032; MA: 0.277 ± 0.033) agreed within 3.8% (Fig. 6D). Further improved model performance could be achieved by adding various subtypes of thyroid cancers to the training data set.

The Fpol method offers several advantages compared to standard FNA analysis. Currently, subjective visual inspection is used to provide a diagnosis based on cytomorphological features in a process that is expensive, labor-intensive, and time-consuming. In addition, diagnostic accuracy of FNA may be impacted by the clinical expertise and experience of the reading cytopathologist. This is especially true for indeterminate cytology and borderline nodules (up to 40% of thyroid FNA cases), where atypical cytomorphology fails to detect malignancy, but prevents a straightforward benign classification. In comparison, Fpol yields conclusive results by classifying thyroid cells into malignant or benign categories based on objective and quantitative measurements. Automation accelerates the process, enabling fast cell segmentations and Fpol calculations, and delivers an accurate diagnosis within seconds. It removes the need for highly trained personnel and substantially reduces costs, creating a path for clinical implementation of automated Fpol technology.

In general, AI systems are not considered trustworthy for autonomous medical applications^49^. To improve safety while still taking full advantage of the data-driven paradigm^50^, one typical approach is human-AI teaming, where the AI system provides input on which a human expert can iterate or decide. This is exactly the setting foreseen for Fpol technology, which can provide standalone evaluations and/or augment conventional cytology in challenging cases. Once integrated into a pathology workflow, the automated method provides verifiable cell segmentations and Fpol assessments that can be interpreted in the context of other diagnostic exams (e.g., blood tests, etc.). The results indicate that the model yields considerable savings in labor and processing time as compared to the alternative manual data processing. Automated Fpol imaging approach holds the potential to reduce the rate of indeterminate thyroid cytology, expensive genetic testing, and unnecessary diagnostic surgeries. Future work includes development of a fail-safe user interface for the technology, plus improved segmentation performance using a larger data set of malignant, benign, and normal thyroid cells.

## Materials and methods

### Study design

We have used data from a previously published, double-blind clinical study with excess, deidentified thyroid tissues^43^ to train and test a deep learning model^51–53^ for rapid, quantitative detection of thyroid cancer at the cellular level. The thyroid specimens were collected from malignant tumors (PTC, FTC, and MTC), benign nodules (FTA and MNG), and normal thyroid epithelial tissues. Training refers to the process of teaching the deep learning model to segment diagnostically relevant thyroid cells based on recognizable features and patterns in the optical images, whereas testing indicates an evaluation of the trained model performance on tasks that included cell selection, segmentation, and Fpol data analysis. The training samples represented a range of pathologically diverse thyroid tissue types with the following diagnoses: PTC, FTA, and normal thyroid cells. Training data included six samples from four subjects that contained 90 images with 850 cells contoured by a human expert. Test samples represented cancerous and non-cancerous thyroid conditions and included the following diagnoses: PTC, FTC, MTC, FTA, MNG, and normal. Test data included seventeen samples from twelve subjects that contained 34 images with 601 cells contoured by the model and/or human expert. The training and test sets were non-overlapping (i.e., did not include the same images or data). For each test sample, we evaluated and compared the number of cells selected, segmented areas, and Fpol values obtained automatically and manually. The manually processed data served as the ground truth. The results of the study were statistically analyzed using the Student’s *t*-test.

All methods were carried out by following relevant guidelines/regulations. The University of Massachusetts Institutional Review Board (IRB) determined that this study did not constitute regulated research as specified by the United States Food and Drug Administration (FDA) and/or Department of Health and Human Services (DHHS), as only excess, deidentified thyroid tissues obtained from surgeries were used. Therefore, the study was exempt from University of Massachusetts IRB approval, and any requirement for patient consent was waived.

### Automated segmentation model

**Figure 7:**
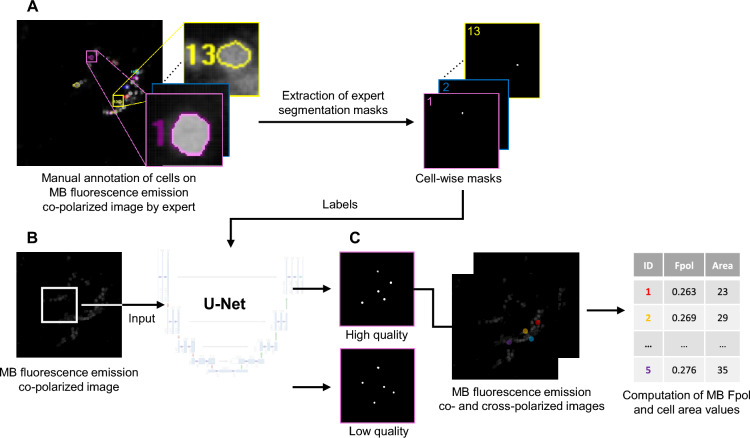
Outline of the segmentation model. (**A**) Ground truth labels of cells based on expert manual annotation were converted into segmentation masks for the AI training. (**B**) Masks and MB fluorescence emission images enter supervised training of the U-Net as labels and input, respectively. (**C**) Algorithm segments the cells and computes the corresponding MB Fpol and cell area values.

We used deep fully convolutional neural networks with U-Net architecture for image segmentation^52,53^, which is particularly suitable for biomedical image analysis, because of its high-resolution processing (Fig. 7). The network is trained fully supervised end-to-end^53,54^. Image segmentation requires a relatively small number of images for training, because each pixel constitutes an example. Previous work showed that the training annotation of the training data can even be sparse (i.e., not all pixels must have a label)^55^. In our task, the input to the network is a pair of averaged co-polarized and cross-polarized MB fluorescence emission images that corresponded to the same field of view. The in-house built, multi-modal confocal imaging system and image acquisition protocol were thoroughly described in previous publications^38,43^. All the images were 8-bit grayscale format, with a file size of 1 MB, field of view of 205 µm^2^ (1000 × 1000 pixels), lateral resolution better than 0.9 µm, and axial resolution of 2 µm. The images were processed with a standard U-Net with five resolution levels (architectural details and training procedure are described in Supplementary Methods). The output produced two segmentation masks: one for cells with high quality signal and another for cells with low quality. Low quality of individual cells was observed, for example, when they were out of focus. The algorithm computed MB Fpol and cell area values for each high quality cell.

### Model training

**Figure 8:**
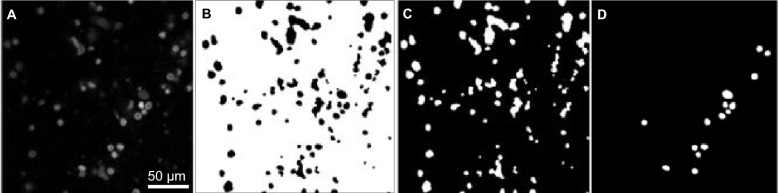
Example data for the network. (**A**) Input co-polarized MB fluorescence emission image. It is an 8-bit, grayscale image (1000 × 1000 pixels). Binary masks of (**B**) background, (**C**) ambiguous cell signal, and (**D**) high-quality cells.

The cytological samples that were set aside as a test set were not used during the training of the network to avoid using the same data once in training and then again during testing^52,53^. The U-Net was trained with a two-channel input (averaged co-polarized and cross-polarized MB fluorescence emission images) and corresponding label masks for diagnostically relevant cells in the images. All label masks in the training set were generated based on annotation masks produced by an expert in optical microscopy and verified by a study pathologist. Figure 8 presents example data for the U-Net. A MB co-polarized fluorescence emission image, utilized as input to the deep neural network, is shown in Fig. 8A. It displays cytomorphological features of the cells (e.g., cell membrane, nuclear size and shape, etc.). Figures 8B–D show binary masks of the background (Fig. 8B), regions labeled as cell-like structures (Fig. 8C), and regions labelled as high quality cells (Fig. 8D). The masks of high-quality cells were drawn by an expert trained in microscopy and pathology, and selected because they were deemed diagnostically relevant. However, the expert did not outline all diagnostically relevant cells, meaning that a lack of a mask over a cell does not imply that the cell is not diagnostically relevant. Furthermore, the distinction between diagnostically relevant and irrelevant cells is not definitive, leading to ambiguity in the labels. We therefore introduced one output for the high-quality cells (those selected by the expert and containing only diagnostically relevant cells) and one for ambiguous cells (those not selected by the expert and therefore containing diagnostically relevant and irrelevant cells). This reduces negative effects of false positive predictions of high-quality cells that resemble ambiguous cells. The cross-entropy loss with equal weight for all outputs was optimized using adaptive moment estimation and batch size of four. We applied online augmentations to reduce overfitting, including rotation, flips, scaling, shearing, elastic transformations, and random cropping.

Model training and testing was performed on a system equipped with an NVIDIA T4 GPU (NVIDIA, Santa Clara, CA) with 16 GB of video RAM, 70 watts of thermal design power, and theoretical 8.1 TFLOPS of peak computing power. The training required up to 8 h. Additional training time would be needed without dedicated hardware, whereas the time would be shorter if a more powerful GPU was used.

### Post-processing

Following the segmentation, and prior to the quantitative analysis, we improved the sensitivity and specificity of the binary segmentation masks with post-processing operations. The pixel-wise probability for foreground and background was binarized with a threshold of 0.5. We corrected some segmentation inconsistencies and increased image clarity through a binary morphological process. We applied a dilation operation with a 3 × 3 kernel in three iterations, which expands segmented regions, thereby resolving small gaps in the objects. Following this, an erosion operation, mirroring the kernel size and iteration parameters of dilation, was performed to revert the dilated areas to their initial boundaries. We eliminated some spurious clusters of pixels identified as cells with a morphological opening operation using an elliptical structuring element with a 15 × 15 kernel size, which offers versatility to accommodate diverse noise shapes.

### Fpol calculations

In the binary segmentation mask, individual high-quality cells were defined and identified via connected component labeling. Within each cell, the average Fpol value was computed. Averaged co-polarized and cross-polarized MB fluorescence emission images were generated, then a threshold was applied to eliminate saturated pixels and background (pixel values > 254 and < 3, respectively). Mean pixel values across the cell area were used to calculate MB Fpol:

1 $$Fpol = (I_{co} - G \cdot I_{cross} )/\left( {I_{co} + G \cdot I_{cross} } \right)$$

where $I_{co}$ and $I_{cross}$ represent averaged co-polarized and cross-polarized fluorescence intensities, respectively. $G$ = 0.75 is the calibration factor of the microscopy system^43^.

### Performance evaluation

For the quantitative evaluation, we analyzed the test set using AU and MA data processing methods. The MA results were obtained previously and represented the ground truth^43^. The test samples were processed using the U-Net, then segmented cells were enumerated via connected component labeling and Fpol values were auto-calculated as described. For each test case, we compared cell selections, total number of selected cells, segmented region areas, and Fpol values obtained automatically with the corresponding parameters from the ground truth. Cells segmented by both AU and MA methods are referred to as *matched* cells. However, some AU selected cells may have no corresponding MA selected cell and vice-versa (i.e., *unmatched* cells). Percent differences in Fpol values or cell areas between AU and MA defined matched cells were calculated as follows:

2 $$\Delta Fpol = \frac{{Fpol_{1} - Fpol_{2} }}{{\left( {\frac{{Fpol_{1} + Fpol_{2} }}{2}} \right)}} \times 100\%$$

3 $$\Delta Area = \frac{{Area_{1} - Area_{2} }}{{\left( {\frac{{Area_{1} + Area_{2} }}{2}} \right)}} \times 100\%$$

where $\Delta Fpol$ and $\Delta Area$ represent the percent differences, and $Fpol_{1}$ and $Fpol_{2}$, and $Area_{1}$ and $Area_{2}$, are the respective quantities obtained for the cell via AU and MA methods, respectively.

Due to different segmentation protocols, cells in the AU and MA data sets may have different region labels (e.g., automatically selected cell AU #1 may correspond to manually selected cell MA #2, etc.).

### Statistical analysis

Statistical analysis was performed to evaluate the significance of differences between the AU and MA processing techniques^56,57^. It tested the null hypothesis that there are significant differences between Fpol values of malignant versus benign cells and malignant versus normal cells, and that there are no significant differences in MB Fpol values derived from AU versus MA processing methods. The cells were organized into six groups: (i) malignant (AU processing), (ii) benign (AU processing), (iii) normal (AU processing), (iv) malignant (MA processing), (v) benign (MA processing), and (vi) normal (MA processing). In addition, eleven indeterminate samples were analyzed separately. They were organized in 4 groups: (i) malignant (AU processing), (ii) benign (AU processing), (iii) malignant (MA processing), and (iv) benign (MA processing). In our previously published work^43^, we demonstrated that Fpol values are not patient specific, therefore we analyzed average Fpol values and corresponding standard deviations obtained for each group. The distribution of the Fpol values across the samples was confirmed to be normal, therefore the paired two-tailed Student’s *t*-tests were utilized. The significance was Bonferroni corrected^57^. The differences were considered statistically significant when *p* < 0.01.

### Patents

Yaroslavsky AN, Patel R, Wirth D. “Devices and Methods for Optical Pathology.” PCT/US2012/025678.

### Institutional review board statement

Not applicable. The Institutional Review Board of University of Massachusetts has determined that this study was not regulated research as defined by DHHS (Department of Health and Human Services) and FDA (Food and Drug Administration) regulations, because only unidentified, excess thyroid tissues from surgeries were used. Therefore, this study was not regulated by the ethic approval committee.

### Supplementary Information

Supplementary Information.

## Data Availability

The experimental data, reference model, and code are available from the corresponding author upon request.
