# Supplementary material for: Deep learning-based cell segmentation for rapid optical cytopathology of thyroid cancer
Source: Sci Rep. 2024 Jul 16;14:16389. doi: 10.1038/s41598-024-64855-2 (PMC11252353; doi:10.1038/s41598-024-64855-2)
Supplement: Supplementary file 1 — Supplementary Information. [file 41598_2024_64855_MOESM1_ESM.pdf]

Supplementary Information for

Deep learning-based cell segmentation for rapid optical cytopathology of thyroid cancer

Peter R. Jermain[1,2], Martin Oswald[3], Tenzin Langdun[3], Santana Wright,[1] Ashraf Khan[4], Thilo Stadelmann,[3,5] Ahmed Abdulkadir,[3,6] and Anna N. Yaroslavsky[1,7]*

[1]Advanced Biophotonics Laboratory, University of Massachusetts Lowell, Lowell, MA, United States
[2]Department of Radiation Medicine, MedStar Georgetown University Hospital, Washington, DC, United States
[3]Centre for Artificial Intelligence, Zurich University of Applied Sciences, Winterthur, Switzerland
[4]Department of Pathology, UMASS Chan Medical School-Baystate, Springfield, MA, United States
[5]ECLT European Centre for Living Technology, Venice, Italy
[6]University Hospital of Old Age Psychiatry and Psychotherapy, University of Bern, Bern, Switzerland
[7]Department of Dermatology, Massachusetts General Hospital, Boston, MA United States
*****Correspondence: Anna_Yaroslavsky@uml.edu

This PDF file includes:

Table S1: PTC case (sample #6)
Table S2: FTA case (sample #7)
Table S3: FTC case (sample #5)
Table S4: MTC case (sample #10)
Supplementary Methods
Table S5: Parameters of the convolutional layers in the analysis and synthesis path of the U-Net.

Abbreviations:

AU – Automated
DNS – Did not segment
Fpol – Fluorescence polarization
FTA – Follicular thyroid adenoma
FTC – Follicular thyroid carcinoma
MA – Manual
MTC – Medullary thyroid carcinoma
N/A – Not applicable
PTC – Papillary thyroid carcinoma

Supplementary Table S1. PTC case (sample #6)

Cell No. (MA)	Cell No. (AU)	Fpol (MA)	Fpol (AU)	Fpol % Difference (MA versus AU)	Cell Area (MA) (μm^2)	Cell Area (AU) (μm^2)	Cell Area % Difference (MA versus AU)
1	3	0.283	0.291	2.7%	22.2	19.9	-11.1%
2	4	0.272	0.285	4.6%	17.8	13.0	-37.2%
3	5	0.259	0.266	2.6%	18.6	18.0	-3.2%
4	6	0.255	0.265	3.8%	14.6	13.5	-8.3%
5	7	0.277	0.280	1.1%	18.6	17.5	-6.5%
6	8	0.254	0.269	5.6%	14.3	9.6	-48.6%
7	9	0.287	0.296	3.0%	21.5	18.0	-19.5%
8	10	0.295	0.308	4.2%	19.3	17.4	-11.1%
DNS	0	N/A	0.282	N/A	N/A	14.7	N/A
DNS	1	N/A	0.257	N/A	N/A	15.5	N/A
DNS	2	N/A	0.288	N/A	N/A	10.0	N/A

Supplementary Table S2. FTA case (sample #7)

Cell No. (MA)	Cell No. (AU)	Fpol (MA)	Fpol (AU)	Fpol % Difference (MA versus AU)	Cell Area (MA) (μm^2)	Cell Area (AU) (μm^2)	Cell Area % Difference (MA versus AU)
1	0	0.103	0.100	-3.0%	50.2	48.9	-2.6%
2	1	0.113	0.116	2.6%	30.3	34.1	11.0%
3	2	0.162	0.150	-8.0%	48.6	60.7	19.9%
4	3	0.170	0.190	10.5%	29.8	23.4	-27.2%

Supplementary Table S3. FTC case (sample #5)

Cell No. (MA)	Cell No. (AU)	Fpol (MA)	Fpol (AU)	Fpol % Difference (MA versus AU)	Cell Area (MA) (μm^2)	Cell Area (AU) (μm^2)	Cell Area % Difference (MA versus AU)
1	DNS	0.276	N/A	N/A	21.3	N/A	N/A
2	10	0.325	0.321	-1.2%	25.1	18.0	-39.6%
3	15	0.284	0.308	7.8%	26.5	17.4	-52.4%
4	0	0.284	0.276	-2.9%	11.2	8.0	-39.4%
5	2	0.281	0.296	5.1%	15.7	12.0	-31.2%
6	5	0.297	0.314	5.4%	15.1	9.7	-55.2%
7	3	0.296	0.309	4.2%	16.0	12.8	-24.6%
8	4	0.296	0.304	2.6%	16.1	14.6	-10.7%
9	7	0.300	0.295	-1.7%	19.5	15.9	-23.2%
10	9	0.318	0.311	-2.3%	21.4	19.6	-9.3%
11	13	0.309	0.299	-3.3%	26.9	21.5	-25.3%
12	12	0.298	0.293	-1.7%	15.2	13.1	-16.2%
13	6	0.297	0.294	-1.0%	17.7	13.1	-34.9%
DNS	1	N/A	0.294	N/A	N/A	7.9	N/A
DNS	8	N/A	0.288	N/A	N/A	10.6	N/A
DNS	11	N/A	0.301	N/A	N/A	15.5	N/A
DNS	14	N/A	0.317	N/A	N/A	15.7	N/A

Supplementary Table S4. MTC case (sample #10)

Cell No. (MA)	Cell No. (AU)	Fpol (MA)	Fpol (AU)	Fpol % Difference (MA versus AU)	Cell Area (MA) (µm^2)	Cell Area (AU) (µm^2)	Cell Area % Difference (MA versus AU)
1	19	0.276	0.275	-0.4%	20.3	19.857	-2.2%
2	18	0.225	0.228	1.3%	24.0	22.42	-7.0%
3	16	0.228	0.262	13.0%	19.2	14.057	-36.7%
4	DNS	0.287	N/A	N/A	29.1	N/A	N/A
5	1	0.264	0.255	-3.5%	26.2	29.27	10.5%
6	DNS	0.308	N/A	N/A	16.0	N/A	N/A
7	DNS	0.230	N/A	N/A	25.8	N/A	N/A
8	DNS	0.256	N/A	N/A	20.3	N/A	N/A
9	2	0.268	0.293	8.5%	28.9	19.563	-47.8%
10	5	0.229	0.242	5.4%	20.7	17.146	-20.6%
11	6	0.281	0.302	7.0%	41.1	39.63	-3.7%
12	8	0.307	0.313	1.9%	53.8	58.541	8.1%
13	7	0.278	0.310	10.3%	27.1	25.698	-5.5%
14	15	0.304	0.327	7.0%	25.83	22.042	-17.2%
15	12	0.335	0.317	-5.7%	41.525	56.208	26.1%
16	13	0.314	0.310	-1.3%	40.45	51.46	21.4%
17	10	0.299	0.316	5.4%	35.375	40.239	12.1%
18	3	0.305	0.300	-1.7%	29.1	30.069	3.2%
DNS	0	N/A	0.246	N/A	N/A	19.878	N/A
DNS	4	N/A	0.241	N/A	N/A	10.926	N/A
DNS	9	N/A	0.311	N/A	N/A	50.493	N/A
DNS	11	N/A	0.308	N/A	N/A	108.719	N/A
DNS	14	N/A	0.333	N/A	N/A	28.703	N/A
DNS	17	N/A	0.314	N/A	N/A	25.53	N/A

Supplementary Methods

We employed a relatively standard U-Net with five resolution levels: first with decreasing resolution in the analysis path and then increasing resolution in the synthesis paths. Each processing block consisted of two 3×3 unpadded convolutions followed by rectified linear activations. The down-sampling in the analysis path was achieved with 2×2 pooling with stride 2×2, and the up-sampling in the synthesis path was obtained with 2×2 up-convolutions with stride 2×2. The features from both paths were put together utilizing a concatenation step. This enabled the network to integrate coarse high-level features with a large spatial context and fine low-level texture information. This architecture resulted in a receptive field of 185 pixels in both spatial directions, which means that for the classification of each pixel a neighborhood of 92x92 pixels around the central pixel is involved. Both outputs, one for high-quality cells and one for low-quality cells, had two output channels and a softmax activation with binary cross-entropy loss. The number of channels in each level is listed in Supplementary Table S5.

Supplementary Table S5: Parameters of the convolutional layers in the analysis and synthesis path of the U-Net

Level	Analysis path	Synthesis path
01 (input/output)	64	64
02	128	128
03	256	256
04	512	512
05	1024	

The input to the network was a crop of size 572×572 pixels. The input image and label were mirrored at the border to include crops covering the entire 1000×1000 image extent. The architecture had 31 million trainable parameters. The network was trained for up to 300 epochs with learning with Adam optimizer and learning rate 0.01 and a batch size of four. The training process on an NVIDIA T4 GPU took about 8 hours.
